# Increased Classical Endoplasmic Reticulum Stress Is Sufficient to Reduce Chondrocyte Proliferation Rate in the Growth Plate and Decrease Bone Growth

**DOI:** 10.1371/journal.pone.0117016

**Published:** 2015-02-18

**Authors:** Louise H. W. Kung, M. Helen Rajpar, Richard Preziosi, Michael D. Briggs, Raymond P. Boot-Handford

**Affiliations:** 1 Wellcome Trust Centre for Cell-Matrix Research, Faculty of Life Sciences, The University of Manchester, Manchester, United Kingdom; 2 Environment, Ecology and Evolution Research Group, Faculty of Life Sciences, The University of Manchester, Manchester, United Kingdom; 3 Institute of Genetic Medicine, Newcastle University, International Centre for Life, Central Parkway, Newcastle Upon Tyne, United Kingdom; UMR CNRS 5242—ENS de Lyon- Université Lyon 1, FRANCE

## Abstract

Mutations in genes encoding cartilage oligomeric matrix protein and matrilin-3 cause a spectrum of chondrodysplasias called multiple epiphyseal dysplasia (MED) and pseudoachondroplasia (PSACH). The majority of these diseases feature classical endoplasmic reticulum (ER) stress and activation of the unfolded protein response (UPR) as a result of misfolding of the mutant protein. However, the importance and the pathological contribution of ER stress in the disease pathogenesis are unknown. The aim of this study was to investigate the generic role of ER stress and the UPR in the pathogenesis of these diseases. A transgenic mouse line (*ColIITg^cog^*) was generated using the collagen II promoter to drive expression of an ER stress-inducing protein (Tg^cog^) in chondrocytes. The skeletal and histological phenotypes of these *ColIITg^cog^* mice were characterised. The expression and intracellular retention of Tg^cog^ induced ER stress and activated the UPR as characterised by increased BiP expression, phosphorylation of eIF2α and spliced *Xbp1*. *ColIITg^cog^* mice exhibited decreased long bone growth and decreased chondrocyte proliferation rate. However, there was no disruption of chondrocyte morphology or growth plate architecture and perturbations in apoptosis were not apparent. Our data demonstrate that the targeted induction of ER stress in chondrocytes was sufficient to reduce the rate of bone growth, a key clinical feature associated with MED and PSACH, in the absence of any growth plate dysplasia. This study establishes that classical ER stress is a pathogenic factor that contributes to the disease mechanism of MED and PSACH. However, not all the pathological features of MED and PSACH were recapitulated, suggesting that a combination of intra- and extra-cellular factors are likely to be responsible for the disease pathology as a whole.

## Introduction

The ability of cells to detect, respond to and survive various stresses is essential for maintaining normal tissue homeostasis. Failure to maintain homeostasis can result in compromised cell function and disease. Endoplasmic reticulum (ER) stress can be triggered by a number of different insults including expression of a mutant protein, energy deprivation, hypoxia, metabolic dysfunction and drugs such as tunicamycin and thapsigargin, which affect N-linked glycosylation and ER calcium ion balance respectively. The ER has evolved the unfolded protein response (UPR) which identifies, selects and eventually rejects misfolded nascent proteins, targeting them for degradation. Therefore, the misfolded protein is prevented from being secreted into the extracellular matrix (ECM) where it could potentially have detrimental effects [1–6]. In addition, exposure to stress can also cause damage to the cell by compromising the function of the ER itself. The UPR functions to attenuate protein translation thereby reducing the load of nascent proteins arriving at the ER, to up-regulate genes involved in ER associated degradation (ERAD) and to up-regulate chaperones, such as BiP (Grp 78) to increase the protein folding capacity of the ER. The classical UPR is mediated through three ER resident transmembrane proteins which enable the cell to sense increasing levels of ER stress: the basic leucine zipper pancreatic ER kinase (PKR)-like ER kinase (PERK); activating transcription factor 6 (ATF6); and inositol-requiring enzyme 1 (IRE1) [1–5]. All three components of the UPR are proposed to be maintained in an inactive state through association with the abundant luminal chaperone BiP (Grp 78).

The ER protein folding machinery and UPR have traditionally been thought of as being particularly important for professional secretory cells, such as pancreatic islets, hepatocytes and antibody synthesising cells. More recently, it has become clear that connective tissue cells, which secrete large amounts of ECM proteins, should also be considered as professional secretory cells [5]. For instance, knockout studies have revealed components of the ER protein folding machinery to be essential for the normal development of cartilage and bone [7–14]. PERK-deficient mice exhibit severe osteopenia, early onset type I diabetes, growth retardation and multiple skeletal dysplasias [8, 11]. Similarly, ATF4-deficient mice exhibit delayed bone formation during embryonic development and reduced bone mass, illustrating that the PERK-ATF4 pathway is required for osteoblast differentiation [9, 13]. Cartilage specific knockout of site-1 protease, which mediates cleavage and activation of ATF6 and other related proteins, results in a severe chondrodysplasia with a complete lack of endochondral ossification [10]. Knockout of BBF2H7, a novel member of the CREB/ATF family that is highly expressed in chondrocytes, also results in a severe chondrodysplasia in mice accompanied by growth plate abnormalities [14]. The fact that bone and cartilage are often the principal tissues affected when key factors of the UPR are perturbed illustrates the reliance of these tissues on an effective control of ER stress.

Endochondral bone formation is a complex, tightly regulated process of chondrocyte proliferation, hypertrophy, ECM synthesis, mineralisation and degradation, and chondrocyte apoptosis [15–17]. Disturbances affecting the highly regulated chondrocyte proliferation in the growth plate can result in the development of a wide range of chondrodysplasias [18–20]. For example, mutations in ECM genes encoding collagen IX, matrilin-3 and cartilage oligomeric matrix protein (COMP) result in multiple epiphyseal dysplasia (MED) and pseudoachondroplasia (PSACH) [21]. MED and PSACH are members of the same bone dysplasia family and symptoms include joint pain and stiffness, short-limbed dwarfism and early-onset of osteoarthritis (OA). In many connective tissue disorders caused by mutations in genes for ECM proteins, the diseases were thought to result from either a defect in the ECM due to the presence of mutant ECM protein or because of deficiency of the wild type ECM protein in the matrix, or a combination of both. Now it is evident another mechanism exists where the misfolding of mutant protein in the ER causes increased levels of ER stress and contributes directly to disease pathogenesis [22–23]. Interestingly, cartilage disorders such as MED, PSACH, metaphyseal chondrodysplasia type Schmid (MCDS) and some forms of the type II collagenopathies have been shown to feature elevated classical ER stress markers and activation of an UPR [5, 22–29].

*In vitro* studies have provided valuable mechanistic insights into the consequences of ECM gene mutations. However, *in vivo* work has enabled us to study the disease mechanisms in a physiological context. A mouse model of MED expressing a human disease-causing knock-in mutation in matrilin-3 (p.V194D) exhibited intracellular retention of the mutant protein, elevated ER stress and activation of an UPR [24]. Mutant mice exhibited growth plate abnormalities including altered chondrocyte morphology and spatial alignment, decreased proliferation and spatially dysregulated apoptosis which collectively resulted in a short-limbed dwarfism [24]. A mouse model of PSACH harbouring the p.T583M mutation (equivalent to human p.T585M) in the C-terminal domain of COMP developed a mild short-limbed dwarfism with poorly organised growth plates and mis-localised ECM proteins [25]. Furthermore, these mutant mice also exhibited decreased chondrocyte proliferation, increased and spatially dysregulated apoptosis and up-regulation of classical UPR markers in the growth plate despite the secretion of mutant COMP into the ECM [25]. ER stress and activation of the UPR has also been associated with a mouse model of platyspondylic lethal skeletal dysplasia, Torrance type (PLSD-T) [27]. A mouse model was generated harbouring a missense mutation in the C-propeptide region of collagen II, p.D1469A. The homozygotes displayed a lethal skeletal dysplasia that resembled PLSD-T patients [27]. Furthermore, mutant collagen II was retained inside chondrocytes, with very little secreted into the ECM. Also, the rough ER was abnormally expanded and ER stress genes Grp 94 and CHOP were both up-regulated [27]. In a similar fashion, another knock-in mouse model harbouring the collagen II p.G1170S mutation also displayed pro-collagen misfolding and retention and evidence of ER stress and UPR activation [28]. Although these models of chondrodysplasias have provided great insights into the disease pathogenesis, a direct link between classical ER stress and disease phenotype still remains to be established in this broad skeletal spectrum. Furthermore, there is a great need to further understand the pathological disease mechanisms responsible to enable us to identify potential therapeutic targets.

In order to functionally characterise the role of classical ER stress in the growth plate, we employed a strategy whereby classical ER stress could be stimulated in the cells that express collagen II, matrilin-3 and COMP independently from an ECM gene mutation. We adapted an approach performed previously [23] and induced the expression of a mutant form of thyroglobulin (Tg^cog^), an ER stress-inducing protein, in proliferative zone chondrocytes under the control of the collagen II promoter [30]. Thyroglobulin is the major secretory glycoprotein of the thyroid glands which normally undergoes homodimerisation in the ER and is then exported and processed during thyroid hormone synthesis. In a mouse model of congenital hypothyroid goiter, newly synthesised Tg^cog^ was unable to homodimerise which led to its retention within the ER [31]. It was later discovered that Tg^cog^ contained a single amino acid substitution, L2263P, which was responsible for the ER storage disease (ERSD) in congenital hypothyroidism [32]. Retention of Tg^cog^ within the ER caused a robust UPR [31]. Here we show that the targeted induction of classical ER stress by the collagen II promoter-driven expression of Tg^cog^ in proliferative zone chondrocytes *in vivo* was sufficient to recapitulate one of the key pathological features associated with MED and PSACH, namely decreased bone growth as a result of decreased chondrocyte proliferation rates, but in the absence of spatially dysregulated apoptosis or alterations in chondrocyte morphology and ECM organisation.

## Materials and Methods

### Ethics statement

All mice used in this study were maintained, handled and sacrificed in strict accordance with UK Home Office regulations (under PPL40/3485) and the provisions of the Animals (Scientific Procedures) Act 1986. The work was approved by the University of Manchester Animal Ethical Review Group.

### Generation of *ColIITg^cog^* transgenic mice and genotyping by PCR and real-time qPCR

The generation of *ColIITg^cog^* transgenic mice was performed in a similar fashion to the generation of a previous *ColXTg^cog^* transgenic line [23]. The 10.5 kb mouse collagen type II (ColII) promoter sequence cloned into pBluescript was a kind gift from Dr Attila Aszodi (LMU, Munich; 30). The 8.5 kb cDNA encoding the *cog* mutant form of Tg, including the start codon, a myc tag and the polyadenylation site, was isolated from a previously described construct (*ColXTg^cog^*; 23) by PmeI digestion and subcloned into an EcoRV site within the 3’ end of the ColII promoter. The construct was then removed from the vector by digestion with BssHII and the DNA purified for pronuclear injection into fertilized oocytes collected from FVB mice, which were subsequently implanted in pseudo-pregnant foster mothers.

The resulting offspring were assessed for the presence of the transgene by PCR between the ColII promoter and the Tg^cog^ cDNA using the following primers: ColIITg^cog^-Gen Forward: 5’-GCACCGTTCTCATGTGCAGG-3’ and ColIITg^cog^-Gen Reverse: 5’-TTCCATCTTCAGAGCACTGG-3’. Founder lines that were positive for the transgene (showing a band at ~360bp) were crossed with C57Bl/6 mice and the resulting offspring were assessed for the expression of the transgene by immunohistochemistry (described below). Mice were bred to homozygosity and defined breeding of wild type (+/+) and homozygote (c/c) mice were maintained on a FVB/N/C57Bl6 mixed background. Heterozygous (+/c) and homozygous (c/c) genotypes were determined by quantitating the relative levels of the transgene to that of type X collagen. Real time qPCR on genomic DNA was used with the following primers: ColIITg^cog^ RT Gen Forward: 5’-CATTCTTGGAGAACGCAGG-3’ and ColIITg^cog^ RT Gen Reverse: 5’-ATGTTGGCTGCTACCAGG-3’ and ColX RT Forward: 5’-CTTCCTGTCAAGCTCATCC-3’ and ColX RT Reverse: 5’-TAGGATTGCTGAGTGCTCC-3’. Wild type genomic DNA was used as a control and a no template control was included to monitor contamination. All reactions were performed in duplicate using a SYBR Green Kit on an ABIPrismTM 7000 sequence detector system (Applied Biosystems Ltd). Only wild type and homozygous animals were subsequently used in this study.

### Growth curves, X-rays and bone length measurements

Mice were weighed at 3, 6 and 9 weeks of age, and these values were used to generate growth curves. Mice at 3, 6, and 9 weeks of age were sacrificed and radiographed using a Flaxitron X-ray specimen radiography system (Flaxitron) and X-ray hyperfilm (GE Healthcare). Individual bone lengths were measured from scanned radiographic images using propriety software (Certus Technology Associates Limited, Exeter, UK). Measurements were analysed by three-way ANOVA full-factorial analysis for statistical significance using the JMP 8 statistical software program.

### Histology

Mice were sacrificed either by cervical dislocation or by carbon dioxide overdose under the provisions of the Animals (Scientific Procedures) Act 1986. The hind limbs were dissected and surrounding soft tissue was removed. Dissected tissue samples were fixed overnight in ice-cold, RNase-free 4% (w/v) paraformaldehyde (PFA) in 1 x DEPC-PBS or 95% ethanol/5% (v/v) acetic acid. Bone samples were decalcified in 0.8 M ethyl-diamine tetracetic acid (EDTA) pH 7.4/4% PFA (for PFA fixed samples) or 0.8 M EDTA pH 7.4 (for ethanol/acetic acid fixed samples). Samples were then rinsed in DEPC-treated water, dehydrated through a standard ethanol gradient, cleared in xylene and embedded in paraffin wax. 5 μm thick sagittal sections were cut using a cool-cut HM 355 S microtome (MicRom), collected on positively charged superfrost slides (VWR) and dried overnight prior to histological staining, immunohistochemistry or *in situ* hybridisation. For haematoxylin and eosin (H&E) staining, sections were dewaxed in xylene, rehydrated through a decreasing ethanol gradient, stained with Shandon Instant Hematoxylin (Thermo Scientific) and 0.1% eosin, dehydrated and cleared in xylene prior to mounting. Images were captured using the Carl Zeiss Axiovision microscope fitted with an Axiocam colour CCD camera and associated Axiovision software.

Measurements of growth plate zone heights were taken in the central part of the growth plate section as previously described [23]. The start of the proliferative zone was defined as the point at which the round, individual cells of the resting zone start to become disc shaped and organised into columns. The start of the hypertrophic zone was defined as the point at which proliferative zone chondrocytes round up and become larger. The end of the hypertrophic zone was defined as the vascular invasion front. For each animal, three separate sections spaced at least 75 μm apart in the growth plate were analysed and the data obtained was averaged. Measurements were analysed by one-way ANOVA for statistical significance.

### Immunohistochemistry (IHC)

IHC was performed on 95% ethanol/5% acetic acid fixed sections unless otherwise stated. Sections from paraffin-embedded joints were first deparaffinised in the xylene and rehydrated in graded ethanol and water. Antigen unmasking for collagen X and collagen II IHC were carried out in 1 mg trypsin (Sigma)/ml of PBS for 12 minutes at room temperature followed by washes in PBS. Antigen unmasking for collagen IX IHC was by incubation with 0.2% bovine testicular hyaluronidase (Sigma) in PBS for 30 minutes. Antigen unmasking for Tg^cog^ and BiP IHC was carried out in citrate buffer pH 6.0 heated to > 85°C for 10 minutes followed by washes in PBS. Quenching of endogenous peroxidase activity was carried out in 3% (v/v) hydrogen peroxide in PBS for 5 minutes. Tissue sections were blocked in PBS containing 2% (v/v) serum derived from the same species in which the secondary antibody was produced, for 1 hour at room temperature, and then incubated with primary antibody at 4°C overnight. Primary antibodies used were collagen X (polyclonal rabbit anti-collagen X against recombinant mouse NC1 domain) diluted 1/500 [23], collagen II (Abcam, ab54236) diluted ½, collagen IX (Calbiochem Ltd) diluted 1/100, thyroglobulin was detected via its myc-tag using an anti-myc mouse monoclonal (Millipore, Cat. No. 05–724) diluted 1/300 using a mouse-on-mouse (M.O.M.) immunodetection kit (Vector Laboratories, BMK2202) and anti-Grp 78 goat polyclonal (Santa Cruz, SC-1051) diluted 1/300. Secondary antibodies used were biotinylated goat anti-rabbit IgG (DakoCytomation Ltd, E0432) diluted 1/1000, biotinylated anti-mouse IgG as provided with the M.O.M. immunodetection kit and biotinylated horse anti-goat IgG (Vector Laboratories, BA 9500) diluted 1/1000. Sections were then incubated with ABC reagent (Vector Laboratories, PK-6100) for 30 minutes and developed using the Vector VIP kit (Vector Laboratories, SK-4600). Slides were dehydrated in increasing concentrations of ethanol then cleared in xylene and mounted using a xylene-based mounting solution. Negative control sections for IHC were performed with the appropriate serum minus the primary antibody. Tissue sections that were known to express the protein of interest were included as positive controls. Images were captured using the Carl Zeiss Axiovision microscope fitted with an Axiocam colour CCD camera and associated Axiovision software.

### *In Situ* Hybridisation (ISH)

DIG-labelled colourimetric ISH was performed as previously described [23]. Briefly, hind limbs were dissected, fixed in ice-cold 4% RNase-free PFA, decalcified and sectioned as described above. The BiP probe was a 350 bp fragment from I.M.A.G.E clone ID 6334883. cDNA probes were cloned into pT7T3, linearised and transcribed using the appropriate restriction enzyme and RNA polymerase respectively.

### Terminal Transferase dUTP Nick End Labelling (TUNEL) Assay

TUNEL assays were performed on PFA-fixed tibial growth plate sections from 3 week old mice using a fluorometric TUNEL kit (Promega, G3250), according to the manufacturers protocol. Briefly, the sections were de-waxed in xylene and rehydrated through decreasing series of ethanol concentrations into water. The sections were immersed in 0.85% NaCl for 5 minutes and washed in PBS. Sections were fixed in 4% PFA in PBS for 15 minutes and washed twice in PBS. To permeabilise the sections and to allow for antigen unmasking, slides were treated with hot (> 85°C) citrate buffer for 10 minutes. The slides were then cooled for 20 minutes before being washed in PBS. The sections were fixed with 4% PFA for 5 minutes and washed in PBS. Sections were incubated with 100 μl equilibration buffer for 10 minutes and then labelled with 50 μl of TdT reaction mix for 1 hour at 37°C. The reactions were stopped by a 15 minute wash in 2 x SSC. Sections were washed in PBS to rinse off excess label. The slides were mounted with Vectashield with DAPI (Vector Laboratories, H1200). Images were collected using the CoolSNAP ES Olympus BX51 camera and associated Metaview software. The green fluorescent apoptotic cells were counted as a percentage of the total number of DAPI stained cells. A cell counting tool in Image J was employed to count the number of cells at the vascular invasion front. Three sections per slide and three slides per mouse were assayed and counted to obtain a reliable estimate of the percentage of apoptotic cells in the growth plate. Nine +/+ mice and eight c/c mice were included in each group. Cell counts were analysed by one-way ANOVA for statistical significance.

### Bromodeoxyuridine (BrdU) labelling

BrdU labelling was performed as previously described [24, 25]. Briefly, 3 week old mice were injected intra-peritoneally with 0.1 ml of cell proliferation labelling reagent (GE Healthcare) per 10 g body weight. Mice were sacrificed 2 hours after injection and dissected tissues were fixed and analysed by IHC using anti-BrdU antibody (1:100, Abcam, ab6326). Antigen unmasking was performed in 4 M HCl for 15 minutes and neutralised with 0.1 M borate buffer. The number of proliferating cells was calculated as a percentage of the total number of cells in the proliferative zone and analysed by one-way ANOVA for statistical significance. Nine sections per growth plate were stained, counted and averaged to obtain representative data for each mouse. Ten +/+ and ten c/c mice were included in each group.

### Western blotting

Protein extracts were obtained as previously described [24, 26]. Briefly, rib cages from 5 day old mice were treated with collagenase (Type 1A, 2 mg/ml) in Dulbecco’s modified Eagle’s medium (DMEM) for 1 hour to remove adherent muscle tissue. The costal cartilage was then dissected from the rib cage and the perichondrium layer removed. The cartilage was then further treated with collagenase for 3 hours to digest away the collagen matrix and release the chondrocytes. The chondrocytes were passed through a 70 μm cell strainer and washed twice with PBS. For western blot analysis, aliquots of 1 x 10^5^ chondrocytes were prepared and resuspended in 25 μl SDS loading buffer containing β mercaptoethanol. These aliquots were boiled and loaded onto SDS-PAGE gels. The gel was electroblotted onto a nitrocellulose membrane and blocked for 1 hour with 3% BSA (w/v) TBST. Primary antibodies against Grp78/BiP (Santa Cruz, SC-1051), phosphorylated eIF2α (Cell Signalling, 9721), total eIF2α (Cell Signalling, 9722) and ATF6 (Imgenex, IMG-273) were diluted 1/500 in blocking solution. Primary antibody against β tubulin (Sigma, T4026) was diluted 1/10,000 in blocking solution. Membranes were then incubated with the appropriate secondary antibody. The membrane was visualised using the Li-Cor Imaging machine and Odyssey Software V2.1.

Western blots on 3 week old rib growth plate extracts were prepared as previously described [23]. Rib growth plate tissue was dissected from 3 week old mice, homogenised and boiled in SDS loading buffer containing β mercaptoethanol and centrifuged. The protein concentration of extracts was assayed using the Pierce bicinchoninic acid (BCA) protein assay kit (Thermo Scientific) with a bovine serum albumin standard curve and samples containing 20 μg were loaded on SDS-PAGE gels and western blotted as above. The blots were quantified by densitometry analysis using ImageJ software and normalised to a loading control. The results were analysed by independent samples t-test for statistical significance.

### PCR analysis to detect *Xbp1* splicing

Rib chondrocytes from 5 day old mice and rib growth plates from 3 week old mice were isolated as described above. The RNA was extracted from chondrocytes and growth plates using Trizol following manufacturer’s instructions. RNA concentration was determined by a Nanodrop ultra-low-volume spectrophotometer. Purified RNA samples of 0.5 μg were subsequently used to synthesize first strand cDNA using the Taqman Reverse Transcription Reagent Kit (Life Technologies) according to manufacturer’s instructions. PCR to detect *Xbp1* splicing was performed on equal quantities of +/+ and c/c cDNA using primers flanking the *Xbp1* ER stress-responsive splice site—F: 5’-GAACCAGGAGTTAAGAACACG—3’ and R: 5’- AGGCAACAGTGTCAGAGTCC—3’.

## Results

### Generation of transgenic mice expressing Tg^cog^ under the ColII promoter

**Fig 1:**
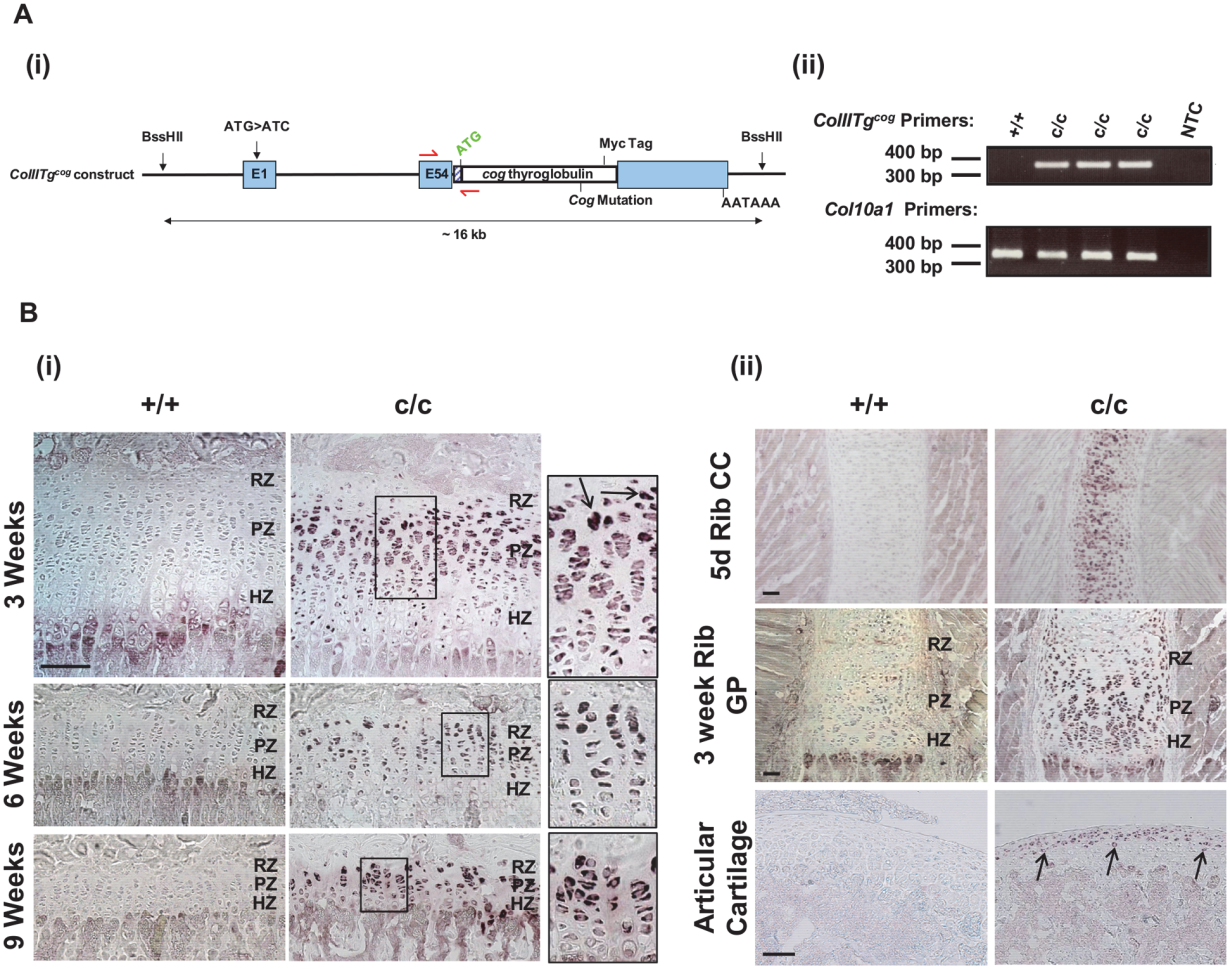
Generation and Tg^cog^ protein expression profile of the *ColIITg^cog^* mouse. (A) (i) Schematic diagram of the *ColIITg^cog^* construct. The collagen II promoter was ligated upstream of the cDNA sequence encoding the myc-tagged *cog* mutant form of thyroglobulin (Tg^cog^). The cloning vector was removed and the construct injected into mouse embryo pronuclei. The annealing sites of the forward and reverse primers used in genotyping to detect the presence of the *ColIITg^cog^* transgene are depicted as red arrows. (ii) A typical genotyping PCR result using the *ColIITg^cog^* primers and *Col10a1* primers as a positive control. (B) IHC for the myc-tagged Tg^cog^ protein on sections from (i) tibial growth plates and (ii) 5 day rib costal cartilage (CC), 3 week rib growth plate (GP) and articular cartilage from mice wild type (+/+) and homozygous (c/c) for *ColIITg^cog^*. The dark staining represents the intracellular accumulation of Tg^cog^ protein (indicated by the arrows) in chondrocytes of c/c mice. The insert is an enlarged section of an area represented by the black box. RZ = Resting zone. PZ = Proliferative zone. HZ = Hypertrophic zone. Scale bar = 100 μm.

The *cog* mutant form of thyroglobulin (Tg^cog^) has previously been shown to stimulate ER stress and activate the UPR due to its intracellular retention and misfolding [23, 31, 33], thereby establishing induction of Tg^cog^ expression as an effective tool with which to induce ER stress *in vivo*. In a previous study, the expression of Tg^cog^ was targeted to hypertrophic chondrocytes through coupling to the collagen X promoter [23]. In the current study, the expression of Tg^cog^ was targeted to proliferative zone chondrocytes as well as other types of chondrocytes through the use of the type II collagen promoter (*ColIITg^cog^*). This expression strategy enabled us to directly test the role of increased ER stress in the disease mechanism of chondrodysplasias, such as MED, PSACH and various type II collagenopathies, independently from the expression of a mutant ECM protein. For easy detection of the Tg^cog^ protein, a myc tag was included at the C-terminus of the transgenic protein (Fig. 1A (i)). Transgenic mice were generated by pronuclear injection. Fourteen founder lines incorporated the transgene allele into the genome, however, only one line expressed the transgene at the protein level and is reported here. Genotyping to distinguish between wild type and transgenic mice was performed by PCR using primers flanking the *ColII-Tg^cog^* junction (Fig. 1A (ii)). Mice wild type and homozygous for the *ColIITg^cog^* transgene are referred to as +/+ and c/c, respectively.

The level of *ColIITg^cog^* transgene expression was evaluated by quantitative real-time PCR. cDNA was generated from RNA extracted from rib chondrocytes of 5 day old +/+ and c/c mice. Quantitative real-time PCR analysis revealed *Tg* expression to be 0.2% the level of endogenous *Col2a1* expression. However, to put this expression level into perspective, in the same samples Tg^cog^ RNA levels were 15 fold higher than *Matn3*, 6 fold higher than *COMP*, equal to *β actin* and 21 fold higher than *GAPDH* (data not shown).

IHC confirmed Tg^cog^ expression and intracellular retention in chondrocytes from c/c mice (Fig. 1B). Tg^cog^ expression was evident in the proliferative and resting zone chondrocytes of the tibial growth plate at 3, 6 and 9 weeks of age (Fig. 1B (i)). Tg^cog^ protein was retained intracellularly within proliferative zone chondrocytes and then decreased in concentration, based on signal intensity, in hypertrophic chondrocytes at all time points examined. To determine if Tg^cog^ expression was directed to chondrocytes other than those in the tibial growth plate, Tg^cog^ IHC was performed on sections from rib and articular cartilage tissue (Fig. 1B (ii)). Tg^cog^ protein was not detected in +/+ rib or articular cartilage but was apparent in the articular chondrocytes and chondrocytes of the rib cartilage of c/c mice in a similar fashion to that of the tibia (and femur—data not shown).

### The expression of Tg^cog^ in chondrocytes caused ER stress in transgenic mice

**Fig 2:**
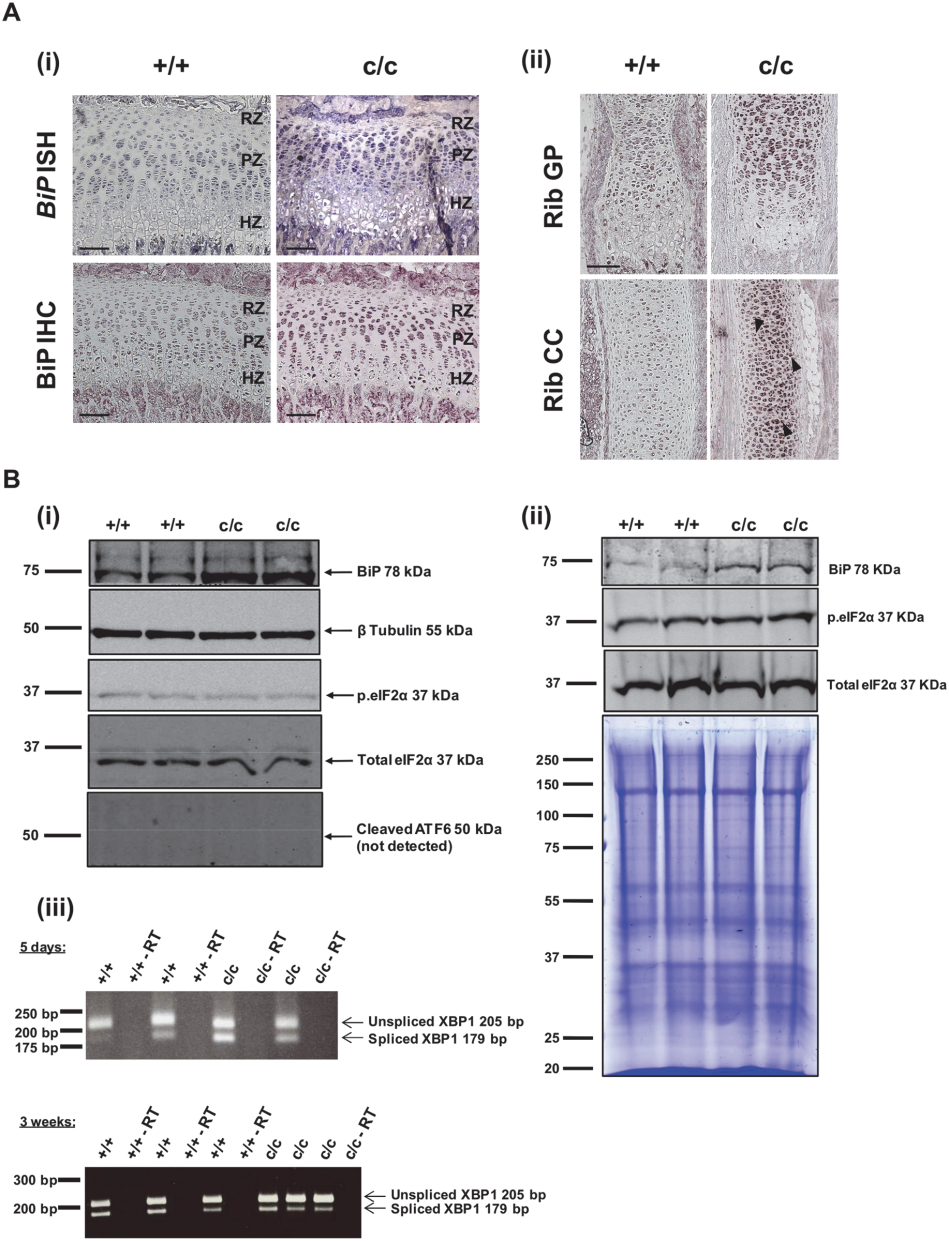
Increased ER stress in the *ColIITg^cog^* mouse. (A) (i) ISH and IHC for BiP in (i) 3 week old tibial growth plate and (ii) rib growth plate (GP) and costal cartilage (CC) sections from +/+ and c/c mice. The presence of the *BiP* mRNA transcript is indicated by the blue staining. The dark purple staining represents the up-regulation of BiP protein in chondrocytes of c/c mice. Scale bar = 100 μm. (B) Western blot analyses of *ColIITg^cog^* mouse rib chondrocytes and growth plate extracts. (i) Chondrocytes were extracted from rib cartilage of 5 days old +/+ and c/c mice. Aliquots of 1 x 10^5^ cells were resolved on SDS-PAGE gels under reducing conditions, and western blotted for BiP, β tubulin, phosphorylated eIF2α (p.eIF2α), total eIF2α and ATF6α. Three replicates of each preparation were performed (not shown). (ii) Western blot analysis of rib growth plates extracts from 3 weeks old +/+ and c/c mice. For each sample, growth plates from the ribs of three 3 weeks old mice were pooled and extracted into SDS-PAGE sample buffer as described in the methods. 20 μg of each extract was resolved on 10% reducing SDS-PAGE gels and western blotted for BiP, phosphorylated eIF2α and total eIF2α. Coomassie staining demonstrates equal protein loading between samples. Each lane represents separate growth plate extracts. (iii) PCR analysis of *Xbp1* splicing in 5 day old rib chondrocytes and 3 week old rib growth plates of +/+ and c/c mice. For each sample, RNA was extracted from 5 day old rib cartilage and 3 week old rib growth plates and pooled from a litter of mice. PCR was performed on cDNA samples using primers specific for sequences flanking the *Xbp1* ER stress-responsive splice site. The 205 bp product represents the unspliced form of *Xbp1* and the 179 bp product represents the spliced form of *Xbp1*. Reactions containing no reverse transcriptase enzyme (-RT) were included as controls.

To confirm that the expression and intracellular accumulation of mutant Tg^cog^ protein in chondrocytes from *ColIITg^cog^* mice triggered an ER stress response, experiments were performed looking at BiP, the major ER chaperone and other ER stress markers including, phosphorylated eIF2α, ATF6α cleavage and activation and *Xbp1* splicing. Using colorimetric ISH and IHC methods, the expression of BiP was markedly increased in tibial growth plate sections from 3 week old c/c mice compared with +/+ mice (Fig. 2A). *BiP* mRNA and protein was clearly up-regulated in the growth plates, particularly in the proliferative zone chondrocytes of c/c mice, indicating activation of an ER stress response. Similarly, an increase in BiP was also evident in the rib growth plate and costal cartilage of c/c mice compared with +/+ mice (Fig. 2A (ii)). To further verify an increase in BiP, western blot analyses were performed on chondrocytes extracted from rib cartilage of 5 day old mice and on rib growth plates from 3 week old mice (Fig. 2B). c/c mice exhibited a significant increase in BiP protein at both 5 days (+/+ 1.00±0.04 (3) vs. c/c 1.97±0.03 (3), mean ± SEM (n), p < 0.001) and 3 weeks of age (+/+ 1.00±0.28 (2) vs. c/c 1.39±0.11 (2), mean ± SEM (n), p < 0.05). The ratio of phosphorylated to total eIF2α (Fig. 2B) was not affected in 5 day rib chondrocytes (+/+ 2.02±0.2 (3) vs. c/c 2.10±0.2 (3)) but was marginally increased in the growth plate extracts from 3 week old c/c mice (+/+ 0.36±0.03 (2) vs. c/c 0.42±0.06 (2)). Furthermore, the cleaved active fragment of ATF6α (50 kDa) was not detectable in either +/+ or c/c chondrocytes (Fig. 2B (i)) or growth plate extracts (data not shown). To examine the IRE1 dependent arm of the ER stress pathway in *ColIITg^cog^* mice, *Xbp1* splicing was examined (Fig. 2B (iii)). The 205 bp and 179 bp products represents the unspliced and spliced forms of *Xbp1* respectively (Fig. 2B (iii). The ratio of the spliced form relative to the unspliced form was higher in 5 day rib chondrocytes from c/c mice (0.7±0.2 (2)) compared with +/+ mice (0.2±0.1 (2)) suggesting activation of the IRE1 pathway in *ColIITg^cog^* mice. However, increased *Xbp1* splicing was not detected in the 3 week rib growth plate extracts (+/+ 0.6±0.1 (3) vs. c/c 0.4±0.1 (3)).

### The expression of Tg^cog^ in chondrocytes caused a mild reduction in growth in transgenic mice

**Fig 3:**
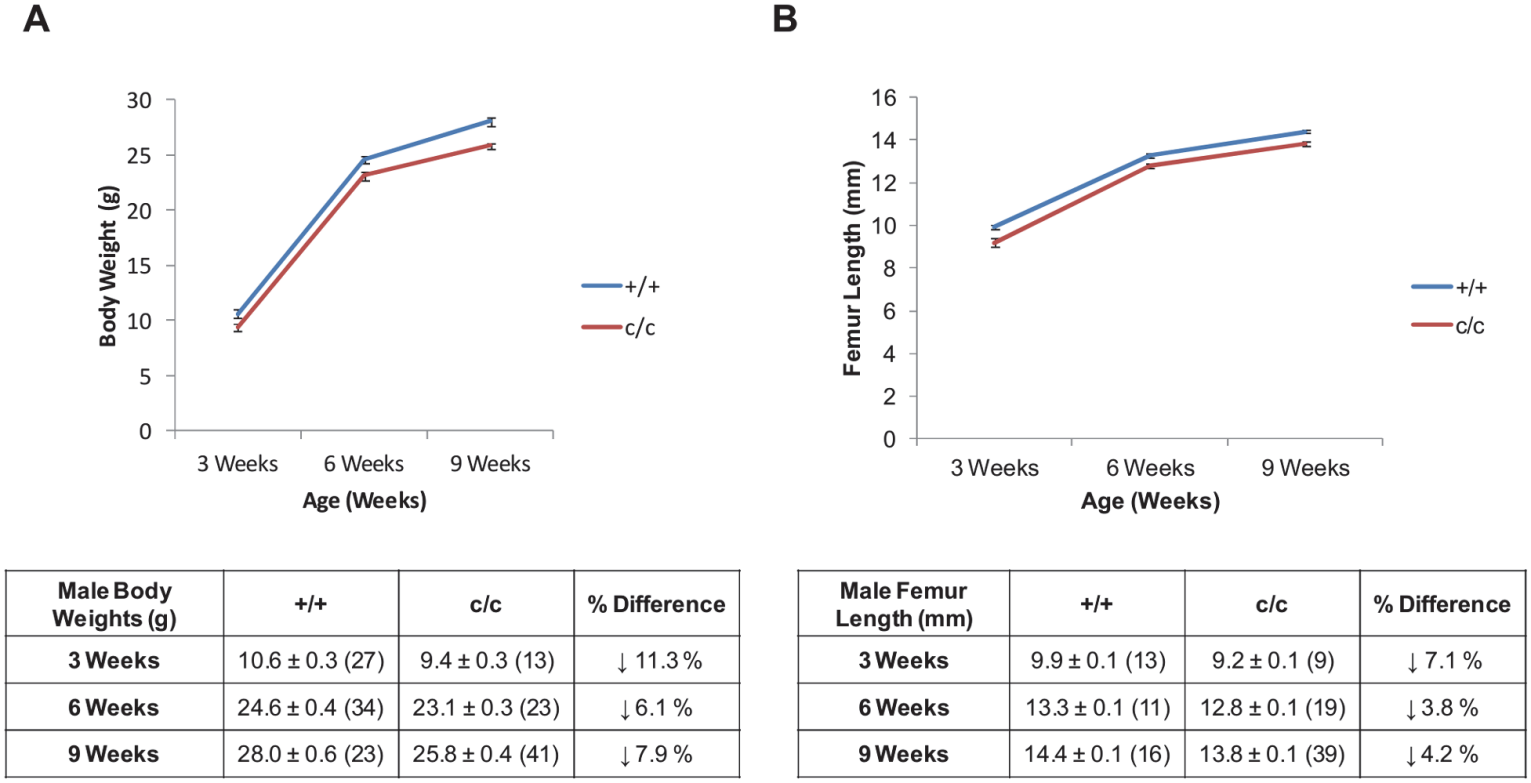
Macroscopic analyses of the *ColIITg^cog^* mouse phenotype. (A) Graph and table of body weights and percentage differences of male +/+ and c/c mice at 3, 6 and 9 weeks of age (mean ± SEM (n)). There was a significant effect of genotype having corrected for age and gender (F_1, 318_ = 1330.2, P < 0.0001). (B) Graph and table of femur lengths and percentage differences between +/+ and c/c male mice at 3, 6 and 9 weeks of age (mean ± SEM (n)). There was a significant effect of genotype having corrected for age and gender (F_1, 186_ = 55.48, P < 0.0001).

Transgenic mice expressing *ColIITg^cog^* were found to be viable with no distinguishing abnormalities. Mice were weighed and x-rayed at 3, 6 and 9 weeks after birth to chart their growth rates over time. From 3 weeks of age, the growth curves for male c/c mice were slightly but significantly reduced compared to +/+ mice (Fig. 3A). Body weights of female c/c mice also showed similar trends (S1A Fig.). The lengths of the femur were used to assess endochondral ossification, whilst the inner-canthal distance (ICD) was used as a measure of intramembraneous ossification. From 3 weeks of age, the femur lengths of male c/c mice were significantly shorter than those of +/+ mice (Fig. 3B). Femur lengths of female c/c mice again showed similar trends (S1B Fig.). The femur lengths of c/c mice were, on average, between 2.3 and 7% shorter than those of +/+ mice over the time course of the experiment (Fig. 3B). Overall, there were no significant differences in the ICD for male and female +/+ and c/c mice at any time point confirming that intramembraneous ossification was not affected (data not shown).

### *ColIITg^cog^* mouse growth plates were normal with no major alteration to cartilage ECM

**Fig 4:**
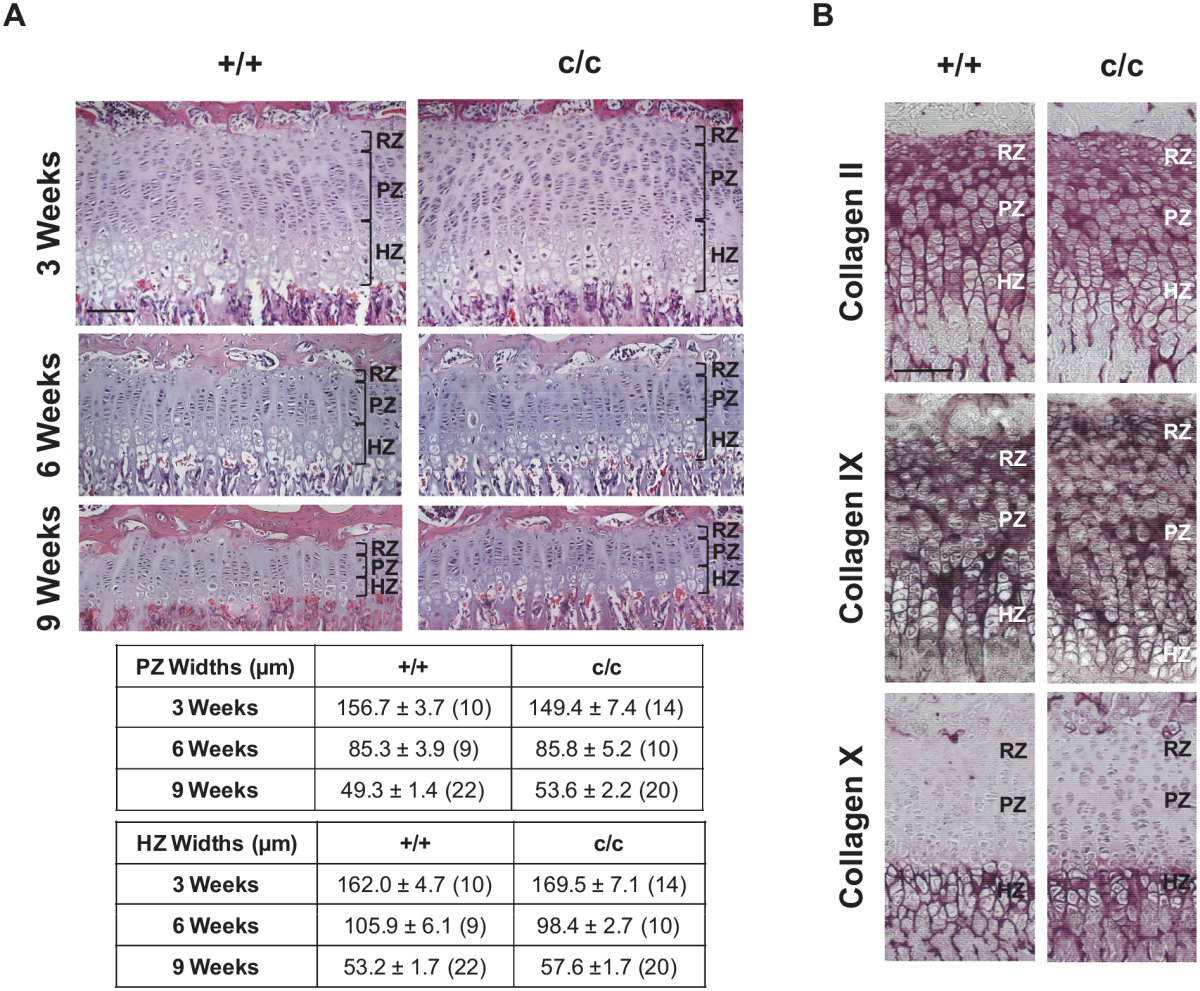
Histological characterisation of the *ColIITg^cog^* mouse growth plate. (A) H&E staining of sections of tibial growth plates from 3, 6 and 9 week old mice wild type (+/+) and homozygous (c/c) for *ColIITg^cog^*. The resting zones (RZ), proliferative zones (PZ) and hypertrophic zones (HZ) are indicated by the black brackets. The table displays width measurements of the PZ and HZ of 3, 6 and 9 week old +/+ and c/c mice (mean ± SEM (n)). There were no significant differences between the PZ and HZ widths of c/c mice versus +/+. (B) IHC for collagen II, IX and X on tibial growth plate sections from 3 week old +/+ and c/c mice. The dark purple staining indicates the collagens localisation within the ECM of the growth plate in +/+ and c/c mice. Scale bar = 100 μm.

H & E staining of sections from the tibial growth plates of +/+ and c/c mice at 3, 6 and 9 weeks of age showed well organised growth plates (Fig. 4A). The resting, proliferative and hypertrophic zones were clearly distinguishable in both +/+ and c/c mice at all time points. The chondrocytes of the proliferative zone were flattened in shape and closely aligned to form compact, ordered columns that were evenly spaced. In +/+ and c/c mice, the proliferative zone chondrocytes underwent hypertrophy, apoptosis and vascular invasion as normal (Fig. 4A). The heights of the proliferative and hypertrophic zones were analysed to detect any expansions or narrowing of the growth plate zones which has been previously seen in other mouse models of chondrodysplasias [23–25]. The widths of the proliferative and hypertrophic zones were unaffected in c/c mice compared with measurements for +/+ mice at all time points examined (Fig. 4A).

To check that the expression and intracellular retention of Tg^cog^ in the ER of c/c mice did not cause any secondary consequences affecting the secretion of matrix proteins, IHC for collagen II, IX and X was performed on sections from 3 week old mice (Fig. 4B). The localisation of collagen II, IX and X were within the ECM of the growth plate in both +/+ and c/c mice (Fig. 4B). The spatial localisations of collagen II, IX and X within the ECM were indistinguishable between genotypes and were not affected by the intracellular retention of Tg^cog^ protein. Furthermore, there were no indications that collagens II, IX and X were co-retained with Tg^cog^ protein.

### The expression of Tg^cog^ in chondrocytes caused a reduction in proliferation in the growth plate of *ColIITg^cog^* mice but did not induce apoptosis

**Fig 5:**
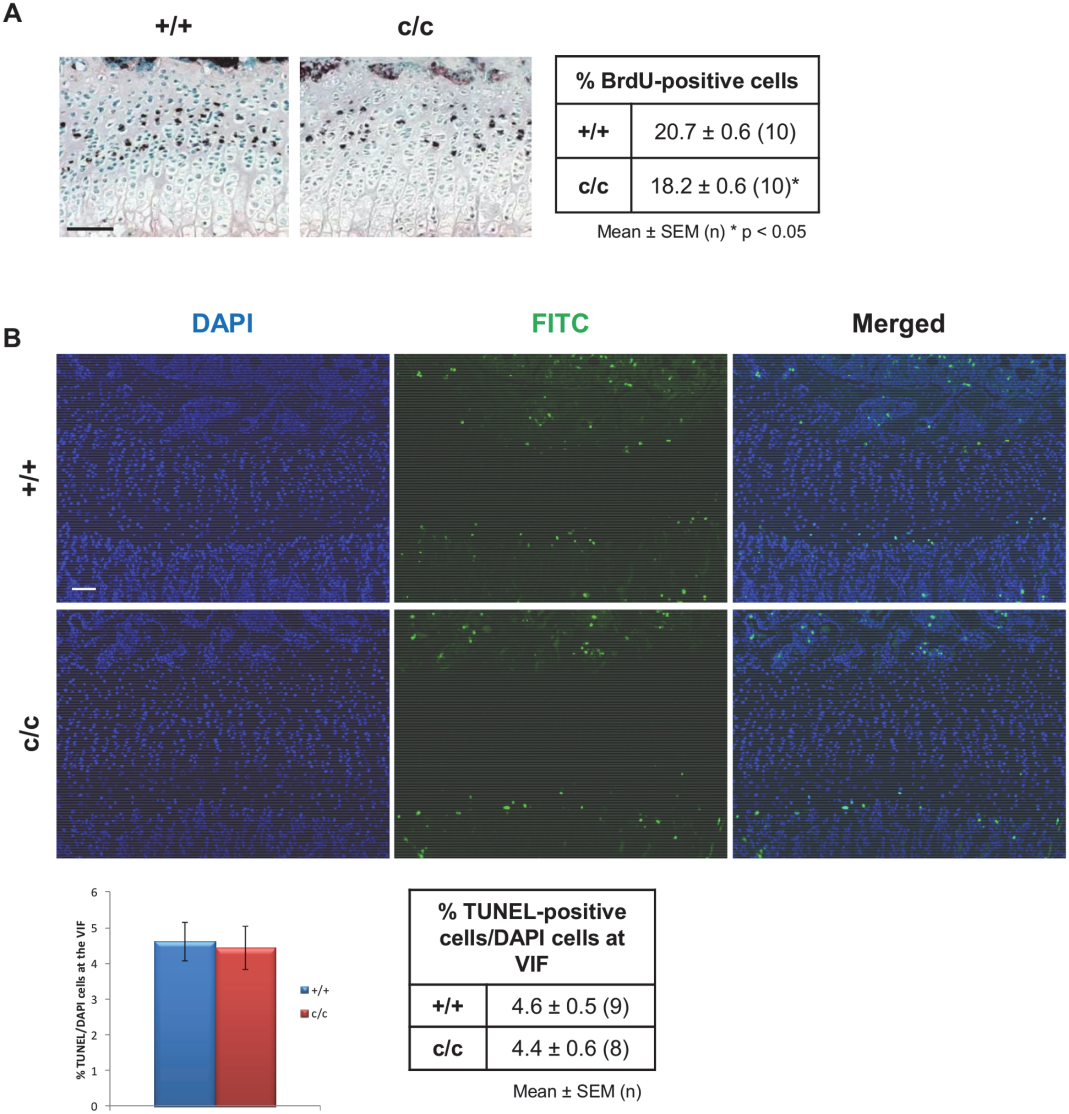
Decreased chondrocyte proliferation but no change in apoptosis in *ColIITg^cog^* mice. (A) 3 week old +/+ and c/c mice were administered with 0.01 ml/g of the nucleotide analogue BrdU 2 hours prior to sacrifice. Representative images of immunostaining for BrdU performed on tibial growth plate sections from +/+ and c/c mice are shown. BrdU positive cells are stained in black. Methyl green staining was used as a nuclear counter-stain. Three sections per slide and three slides spaced at least 75 μm apart were stained, counted and analysed. The table shows the percentage of BrdU-labelled nuclei calculated against the total number of cells in the proliferative zone. c/c mice had significantly lower proliferation rates (by 12%) when compared with +/+ (one-way ANOVA, mean ± SEM (n), * p < 0.05). (B) End-stage apoptosis (DNA fragmentation) was measured in the tibia of 3 week old +/+ and c/c mice using the DeadEnd fluorometric TUNEL system. Representative images of the tibial growth plates showing DAPI and FITC (TUNEL-positive) stained sections are shown. The relative levels of apoptosis were calculated by comparing the number of apoptotic chondrocytes (FITC-labelled nuclei) with the total number of chondrocytes (DAPI-labelled nuclei & FITC-labelled nuclei) at the vascular invasion front (VIF). Scale bar = 100 μm.

The rate of chondrocyte proliferation in the growth plate was determined in 3 week old +/+ and c/c mice by performing BrdU labelling experiments (Fig. 5A). Within the proliferative zone of +/+ mice, 20.7 ± 0.6% (10) (mean ± SEM (n)) of total cells were labelled with BrdU within a 2 hour period, whereas a significantly reduced proportion of cells (18.2 ± 0.6% (10), p < 0.05) were labelled with BrdU in c/c mice (Fig. 5A). This demonstrates a 12% decrease in the rate of proliferation in c/c growth plates at 3 weeks of age.

To determine if increased ER stress, induced by Tg^cog^ expression in chondrocytes, was having a detrimental effect on cell viability the relative level of apoptosis in the growth plate was determined by performing a TUNEL assay on sections from 3 week old +/+ and c/c mice (Fig. 5B). Apoptosis was only apparent at the vascular invasion front (VIF) in +/+ and c/c mice (Fig. 5B). The c/c mice did not exhibit spatially dys-regulated apoptotic cells in the resting or proliferative zones of the growth plate, as seen previously in mouse models of MED and PSACH [24, 25]. In addition, the levels of apoptosis at the VIF in +/+ and c/c mice were comparable (mean ± SEM (n); 4.6 ± 0.5% (9) and 4.4 ± 0.6% (8)) respectively.

## Discussion

Elevated ER stress and activation of a resulting UPR is increasingly recognised as a common feature of a number of connective tissue disorders including various forms of chondrodysplasias [5, 29]. However, the importance of ER stress in the pathogenesis of most of these diseases remains to be established conclusively. Only in the case of MCDS has the direct role of classical ER stress and the resulting UPR been highlighted through studying the effects induced by the targeted expression of Tg^cog^ in hypertrophic chondrocytes which replicated the disease phenotype (*ColXTg^cog^* mouse; 23). Tg^cog^ was chosen as the ER stress-inducing protein based on its efficient intracellular retention and the robust UPR induced by its expression [23, 31–33]. In addition, the targeted expression of Tg^cog^ in hypertrophic chondrocytes resulted in an UPR similar to that induced by mutant collagen X [23].

In this current study, the expression of Tg^cog^ was targeted to proliferative zone chondrocytes as well as other types of chondrocytes through the use of the type II collagen promoter (*ColIITg^cog^*). The induction of classical ER stress in proliferative zone chondrocytes produced both decreased bone growth and reduced chondrocyte proliferation, which are related and important pathological features of MED and PSACH. The targeted ER stress through expression of Tg^cog^ in proliferative zone chondrocytes resulted in the up-regulation of BiP chaperone levels as indicated by western blots on 5 day old rib chondrocytes (Fig. 2B(i)) and 3 week old rib growth plates in c/c mice (Fig. 2B(ii)). ISH and IHC performed on sections of the tibial growth plates revealed increased BiP expression to be most apparent in the proliferative zone chondrocytes (Fig. 2A). These are the same chondrocytes most affected by ER stress in models of MED and PSACH [24, 25]. In order to biochemically detect ER stress markers in this mouse model, we have analysed 5 day rib chondrocytes and 3 week rib growth plate extracts. Rib chondrocytes from 5 day old mice express the Tg^cog^ construct (Fig. 1), provide a reasonable amount of material per mouse but have the limitations that they take several hours to digest from the extracellular matrix prior to extraction and are not growth plate in origin. They therefore provide reasonable insight into the more stable ER stress responses such as *Xbp1* splicing or BiP steady state levels but are not necessarily a good model in which to study markers that can change rapidly, such as, phosphorylated eIF2α. In contrast, 3 week rib growth plates, which are extracted as soon as dissected from the ribs, provide a more reliable snap shot of ER stress markers that may rapidly change but are a mixed population of chondrocytes (resting, proliferative and hypertrophic chondrocyte) some of which do not express the Tg^cog^ transgene. Nevertheless, modest activation of downstream UPR markers, such as phosphorylated eIF2α (3 week growth plate) and spliced *Xbp1* (5 day rib chondrocytes) were apparent in c/c mice accompanied by significant increases in BiP chaperone levels. This modest induction of an increased level of ER stress through the expression of Tg^cog^ in proliferative chondrocytes was sufficient to cause a significant reduction in chondrocyte proliferation which ultimately led to reduced bone growth and illustrates the importance of ER stress in modulating the bone growth rates *per se*. It is important to highlight the fact that the induction of ER stress did not alter matrix protein secretion, the highly organised columnar structure of the growth plate or induce apoptosis. Therefore, increased ER stress in its own right was sufficient to reduce bone growth, signifying the contribution of ER stress in the pathogenesis in this chondrodysplasia phenocopy. The fact that increased ER stress in the *ColIITg^cog^* mouse, independent of an ECM gene mutation, did not fully recapitulate the MED/PSACH disease phenotype demonstrates that other factors, in addition to ER stress, are likely to be involved and the disease mechanisms responsible for the overall phenotype are much more complex than originally thought. A combination of factors such as the ER stress-inducing capacity of each misfolded protein, the effect on matrix secretion and integrity, the disruption to growth plate architecture and the impact on proliferation and apoptosis, all have the potential to contribute to the overall disease mechanism responsible for MED/PSACH.

Mouse models of chondrodysplasias resulting from proliferative zone defects have featured significant increases in the expression of ER stress markers (reviewed in [5] and [29]). For example, the cellular response to the expression of *Matn3* p.V194D has been previously characterised and included increased expression of the chaperones BiP and Grp94 [24, 26]. Interestingly, as with the *ColIITg^cog^* mouse, *Matn3* p.V194D mice did not exhibit ATF6α cleavage and activation despite the high levels of intracellular retention of mutant matrilin-3 [24, 26]. Nevertheless, biochemical or microarray-based evidence for activation of the PERK and IRE1 pathways was apparent in these mouse models (Fig. 2B and [26]). In addition, mice expressing the PSACH-causing *COMP* p.T583M mutation also exhibited biochemical evidence of a mild UPR including phosphorylated eIF2α, cleavage of ATF6α and elevated CHOP expression, accompanying the slight but significant increases in BiP and Grp94 chaperone levels [25]. In this case, the mutant COMP was not retained intracellularly but secreted into the ECM [25], whereas the most frequent COMP mutation (p.D469del) was found to be retained intracellularly, but did not induce a transcriptional UPR [34–37]. Clearly therefore, the induction of ER stress is not directly related to the level of intracellular accumulation of mutant protein, as noted previously in mouse models of MCDS [38]. Furthermore, the degree of UPR activation is dependent upon the nature of the protein, dynamics of protein misfolding and ER stress-inducing capability of the misfolded protein being expressed. To add further complexity to the issue, not only does each disease model exhibit varying levels of ER stress, they also show various degrees of matrix dysfunction due to the lack of matrix protein secretion and/or the presence of a mutant matrix protein in the ECM.

Mice expressing the MED-causing *Matn3* p.V194D mutation demonstrated a high level of mutant matrilin-3 retention. However, a small proportion of mutant protein was also secreted and incorporated into the ECM [24]. The presence of the mutant protein in the ECM could potentially exert a dominant-negative (antimorphic) effect independently from the ER stress and contribute to an ECM dysfunction [39]. It is noteworthy that expression of Tg^cog^ in the *ColIITg^cog^* mouse reported here did not cause any generalised secretion defect as wild type collagens II, IX and X synthesised in the same cells appeared to be secreted normally with no evidence of intracellular retention (Fig. 4B). Additionally, the extractability of structural ECM components from cartilage of a similar transgenic mouse model expressing the apoptosis-inducing G2320R mutant form of Tg under the collagen II promoter (*ColIITg^rdw^*) was shown to be unaltered compared to wild type mice [40]. Therefore, generalised intracellular retention of ECM proteins and altered organisation and integrity of the cartilage ECM as a consequence of Tg^cog^ retention is unlikely to play a pathogenic factor in this study. In mouse models of PSACH, the spatial localisation of key structural matrix proteins (COMP, matrilin-3, collagen IX, aggrecan, collagen II and collagen X) were also disrupted [25, 37], thus potentially altering the structural integrity of the matrix and disrupting signalling events responsible for the alignment of newly divided chondrocytes [25, 34–37]. Therefore, in addition to the robust UPR, the presence of mutant COMP protein within the ECM coupled with the selective retention of COMP binding proteins, in some cases, may act as an additional pathogenic factor in PSACH. The disruption to ECM organisation and integrity of the cartilage matrix are likely to impact on the highly organised structure of the chondrocytes in the growth plate, further influencing the rate of bone growth.

The rate of longitudinal bone growth results from the introduction and arrangement of new chondrocytes in the proliferative zone, the 4–10 fold increase in cell volume during chondrocyte hypertrophy and the synthesis and degradation of matrix components throughout the growth plate [41]. c/c mice displayed clearly distinguishable resting, proliferative and hypertrophic zones of the growth plate (Fig. 4A). Moreover, chondrocytes within the proliferative zone of +/+ and c/c mice adopted a flattened appearance and were able to align properly to form compact columns consisting of 4- and 8-cell chondrons (Fig. 4A). In contrast, the proliferative zone chondrocytes in *Matn3* p.V194D mice adopted a wedged shaped appearance that prevented them from forming compact columns [24]. As a consequence the overall morphology of the growth plate was disrupted. Mouse models of PSACH also exhibit a disrupted columnar arrangement of proliferative chondrocytes [25, 34–37]. Recently, the role of apoptosis and induction of oxidative stress in MED and PSACH has been demonstrated through the generation and characterisation of a transgenic mouse model expressing an apoptosis-inducing G2320R mutant form of Tg, *ColIITg^rwd^* [40]. Interestingly, some of these mice also exhibited dysplastic growth plates with areas of hypocellularity and grossly disorganised columns. [40]. These comparisons demonstrate that classical ER stress alone as seen in the *ColIITg^cog^* mouse reported here, was not sufficient to induce a growth plate dysplasia, a key feature associated with MED/PSACH. The lack of growth plate dysplasia in the *ColIITg^cog^* mice partly explains the lesser effect on bone growth when compared with the *Matn3* p.V194D and *ColIITg^rwd^* mouse models (Table 1).

**Table 1:** Comparison of the *ColIITg^co^* ^*g*^ phenotype with mouse models of MED, PSACH and MCDS.

**Mouse**	**ER Stress (↑ BiP)**	**Mutant ECM Protein Secretion**	**Growth Plate Abnormality**	**BrdU**	**TUNEL**	**Bone Length**
*ColIITg^cog^*	Yes 5 days: 2-fold (protein) 3 weeks: 1.4-fold (protein)	No	No	↓ 12%	No change	↓ 6–7%
*Matn3* p.V194D (MED)	Yes 5 days: 1.4-fold (protein) 3 weeks: 1.3-fold (protein)	Yes	Yes	↓ 16%	Spatially dys-regulated	↓12.5%
*COMP* p.T583M (PSACH)	Yes 2.8-fold (mRNA)	Yes	Yes	↓ 24%	↑ 3.3 fold VIF; ↑ 12 fold PZ; ↑ 2.5 fold RZ	↓ 4% (9 weeks)
*COMP* p.D469Del (PSACH)	No	Yes	Yes	↓ 17%	↑ 5 fold Spatially dysregulated	↓ 6–7% (9 weeks)
*ColIITg^rdw^*	Yes 5 days: 2-fold (protein)	No	Yes	↓ 21%	No change	↓ 8.8%
*ColXTg^cog^* (MCDS)	Yes 2 weeks: 4–6-fold (mRNA)	No	Yes	N/A	N/A	↓ 6%
*Col10a1* p.N617K (MCDS)	Yes 2 weeks: 4–8-fold (mRNA)	Yes	Yes	No change	No change (1 Week)	↓ 15.6%

Phenotypic comparisons between the *ColIITg* ^*cog*^, MED-causing *Matn3* p.V194D (24, 26), PSACH-causing *COMP* p.T583M (25) and p.D469del (37), *ColIITg^rdw^* (40), MCDS-causing *Col10a1* p.N617K and *ColXTg^cog^* mouse models (23, 33). Presence of ER stress was determined by evidence of increased levels of BiP (protein or mRNA). Mutant ECM protein secretion refers to any evidence showing secretion of mutant protein into the ECM contributing to an ECM dysfunction. Growth plate abnormalities include any dysplastic or disorganised growth plate architectures. All comparisons were made at 3 weeks of age unless stated otherwise. N/A—Not applied. VIF—Vascular invasion front; PZ—Proliferative zone; RZ—Resting zone.

The targeted induction of ER stress in proliferative zone chondrocytes caused a 12% decrease in the rate of chondrocyte proliferation of c/c mice at 3 weeks of age and no changes in apoptosis were apparent (Fig. 5). In comparison, 3 week old *Matn3* p.V194D mice displayed a decrease of 16% in the rate of chondrocyte proliferation and spatially dys-regulated apoptosis in the growth plate [24]. A transgenic mouse model of PSACH over-expressing the *COMP* p.D469del mutation exhibited an 11.5% increase in the rate of apoptosis [35]. In comparison, a more physiologically relevant model expressing a knock-in *COMP* p.D469del mutation displayed a 5-fold increase in apoptosis (37; Table 1). Three week old *COMP* p.T583M mice exhibited a 24% decrease in the rate of chondrocyte proliferation and increased apoptosis at the VIF (3-fold), proliferative zone (12-fold) and resting zone (2.5-fold) (25; see Table 1). *ColIITg^rwd^* mice exhibited a 21% decrease in chondrocyte proliferation rate (40). Such comparisons illustrate that even though increased ER stress in its own right in the *ColIITg^cog^* mouse was sufficient to reduce chondrocyte proliferation, the effect was milder than that observed for the *ColIITg^rdw^*, *Matn3* p.V194D, *COMP* p.D469del and *COMP* p.T583M models (Table 1).

All the factors discussed above could have different potentials to hinder bone growth and so far it has been unclear which factor(s) contribute the most to the overall disease pathology. *ColIITg^cog^* mice exhibited a 6–7% reduction in bone length at 3 weeks of age (Fig. 3). In comparison, at the same age, mice homozygous for the *Matn3* p.V194D mutation displayed a 12.5% reduction in bone length (24; see Table 1 for comparisons). Mice homozygous for the *COMP* p.T583M mutation or p.D469del mutation displayed a 4% and 6–7% reduction in bone length respectively, both only apparent at 9 weeks [25, 37]. The *ColIITg^rwd^* mice displayed an 8.8% reduction in bone length at 3 weeks of age [40]. Of course, we cannot rule out the possibility that some of these differences between mouse models may be due to genetic background and/or differences in researcher analyses [42]. However, such comparisons demonstrate that based on bone growth, the *ColIITg^cog^* mouse dwarfism is milder than that seen in the *Matn3* p.V194D and *ColIITg^rwd^* mice but apparently greater than that seen in the *COMP* p.T583M mouse at 3 weeks of age (Table 1). The combined effects of a disrupted growth plate morphology, a greater decrease in proliferation, dys-regulated apoptosis and a potentially defective ECM in the *Matn3* p.V194D mouse could, therefore, explain the greater effect on bone growth in comparison to the *ColIITg^cog^* mouse (Table 1). The affects on chondrocyte proliferation, apoptosis and ECM abnormalities in 3 week old *COMP* p.T583M mice were far greater than those described for *ColIITg^cog^* and *Matn3* p.V194D mice yet with little additional impact upon bone growth (Table 1). Therefore, the comparatively low impact on bone growth reported in 3 week old *COMP* p.T583M mice is surprising and difficult to explain given the severe growth plate phenotypes of these mice. Defining precisely how the growth plate phenotype translates into a bone growth rate remains a challenge in this field. In all these cases the overall pathological effect will be determined by a combination of factors—expression profile with age (i.e. *Col2a1* promoter Vs *Comp* Vs *Matn3*), nature of the mutant protein and the intracellular and extracellular consequences of the expression of mutant ECM protein.

Generation of a transgenic mouse model expressing an apoptosis-inducing G2320R mutant form of Tg, *ColIITg^rdw^* resulted in replication of some of the pathological features of MED and PSACH [40], in a similar but more severe fashion than reported here for the *ColIITg^cog^* mouse. *ColIITg^rdw^* mice exhibited a 2-fold increase in BiP levels [40], comparable to *ColIITg^cog^* mice reported here. In addition, *ColIITg^rdw^* mice also showed an increase in PDI and ERp72 (PDIA4) expression which is not mirrored in *ColIITg^cog^* mice (data not shown). Therefore, activation of certain genes and pathways may be mutation-specific, something that is also reflected when comparing different mutations in COMP (Table 1). Furthermore, *ColIITg^rdw^* mice displayed a 21% reduction in chondrocyte proliferation rate and an 8.8% reduction in bone growth, much more severe than reported for the *ColIITg^cog^* mice here—12% and 6–7% respectively. The differences in cellular response displayed between the two transgenic mouse models may be due to a direct effect of different mutations or perhaps a difference at the level of transgene expression thus explaining the difference in phenotype severity. These allelic series of novel transgenic mouse models demonstrate the significance of classical ER stress and other forms of intracellular stress, such as oxidative stress, in the pathogenesis of chondrodysplasias in this disease spectrum. Although, these transgenic models do not fully recapitulate the disease phenotype of knock-in models of MED and PSACH, nevertheless, they have allowed us to delineate the relative contributions of intracellular and extracellular disease mechanisms to the disease pathogenesis.

In summary, the induction of classical ER stress in proliferative zone chondrocytes produced decreased chondrocyte proliferation and bone growth, both of which are key pathological features of MED, PSACH and diseases associated with type II collagenopathies. The phenotype of the *ColIITg^cog^* mouse demonstrates mechanistically the central role played by classical ER stress in the MED and PSACH disease process. However, the induced ER stress was not sufficient to replicate all the pathological features of MED and PSACH, suggesting that a combination of pathogenic factors including the presence of a defective cartilage ECM in addition to increased ER stress are likely to contribute to disease pathogenesis [39]. Establishing the link between the extracellular and pathological consequences of the presence of mutant ECM protein, separate from the intracellular mechanisms requires further investigation. Nevertheless, strategies aimed at restoring homeostasis in the ER and enhancing proliferation during times of ER stress are likely to be of potential therapeutic benefit in this disease spectrum.

## Supporting Information

S1 Fig Macroscopic analyses of the *ColIITg^cog^* mouse phenotype (female mice). (A) Graph and table of body weights and percentage differences of female +/+ and c/c mice at 3, 6 and 9 weeks of age (mean ± SEM (n)). There was a significant effect of genotype having corrected for age and gender (F_1, 318_ = 1330.2, P < 0.0001). (B) Graph and table of femur lengths and percentage differences between +/+ and c/c female mice at 3, 6 and 9 weeks of age (mean ± SEM (n)). There was a significant effect of genotype having corrected for age and gender (F_1, 186_ = 55.48, P < 0.0001).
(PPTX)
