# Supplementary material for: Increased Classical Endoplasmic Reticulum Stress Is Sufficient to Reduce Chondrocyte Proliferation Rate in the Growth Plate and Decrease Bone Growth
Source: PLoS One. 2015 Feb 18;10(2):e0117016. doi: 10.1371/journal.pone.0117016 (PMC4334961; doi:10.1371/journal.pone.0117016)
Supplement: S1 Fig — (A) Graph and table of body weights and percentage differences of female +/+ and c/c mice at 3, 6 and 9 weeks of age (mean ± SEM (n)). There was a significant effect of genotype having corrected for age and gender (F1, 318 = 1330.2, P < 0.0001). (B) Graph and table of femur lengths and percentage differences between +/+ and c/c female mice at 3, 6 and 9 weeks of age (mean ± SEM (n)). There was a significant effect of genotype having corrected for age and gender (F1, 186 = 55.48, P < 0.0001). (PPTX) [file pone.0117016.s001.pptx]

## Slide 1
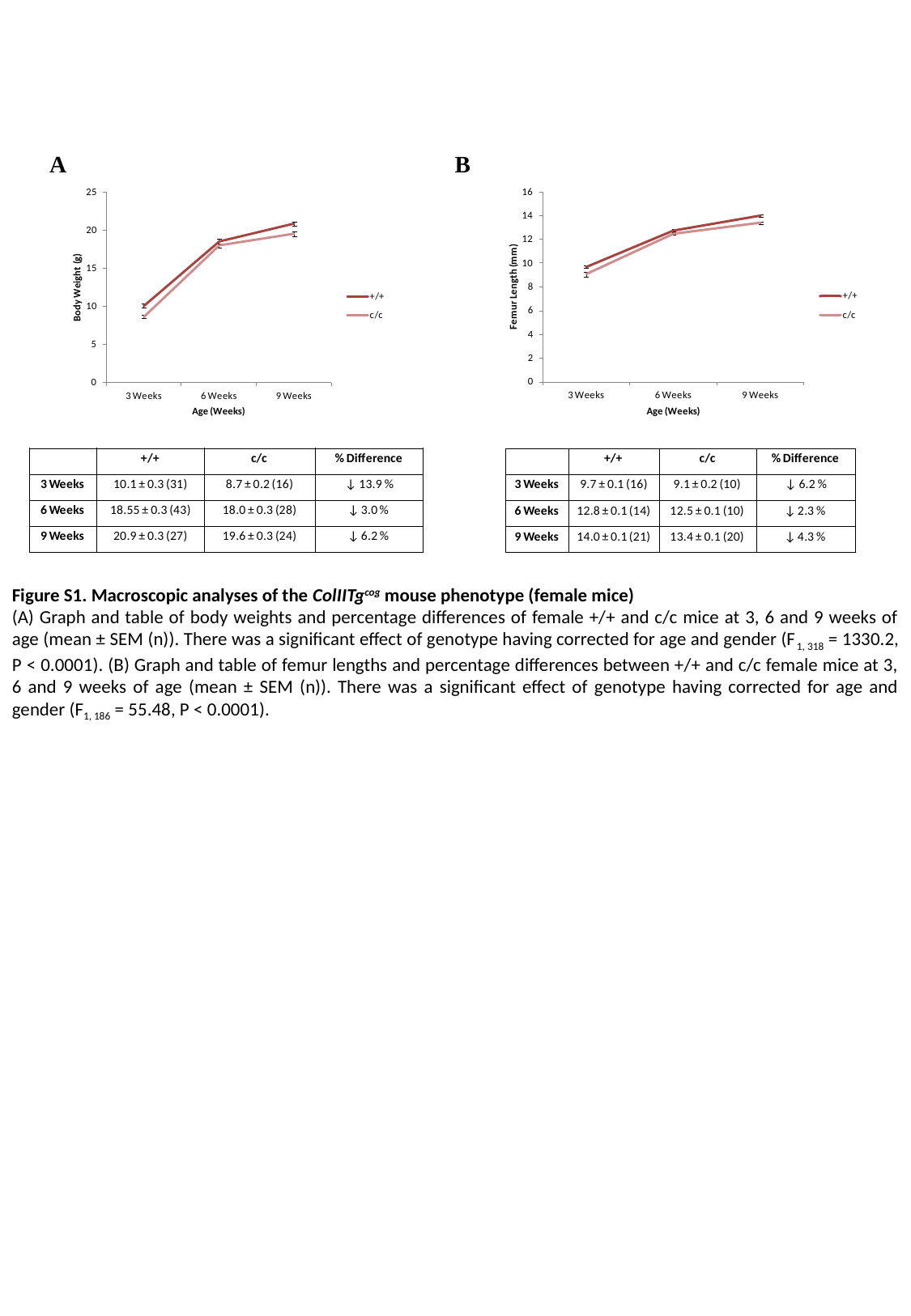

A
B
Figure S1. Macroscopic analyses of the ColIITgcog mouse phenotype (female mice)
(A) Graph and table of body weights and percentage differences of female +/+ and c/c mice at 3, 6 and 9 weeks of age (mean ± SEM (n)). There was a significant effect of genotype having corrected for age and gender (F1, 318 = 1330.2, P < 0.0001). (B) Graph and table of femur lengths and percentage differences between +/+ and c/c female mice at 3, 6 and 9 weeks of age (mean ± SEM (n)). There was a significant effect of genotype having corrected for age and gender (F1, 186 = 55.48, P < 0.0001).
